# Plasma microRNA-143 and microRNA-145 levels are elevated in patients with left ventricular dysfunction

**DOI:** 10.1007/s00380-024-02410-9

**Published:** 2024-05-08

**Authors:** Hirotaka Murase, Shingo Minatoguchi, Kazuki Heishima, Shinji Yasuda, Atsushi Satake, Ryo Yoshizumi, Hisaaki Komaki, Shinya Baba, Shinsuke Ojio, Toshiki Tanaka, Yukihiro Akao, Shinya Minatoguchi, Hiroyuki Okura

**Affiliations:** 1 Department of Cardiology, Gifu Municipal Hospital, 7-1 Kashimachou, Gifu, 500-8513 Japan; 2 Department of Cardiology, Gifu University Graduate School of Medicine, Gifu, Japan; 3 United Graduate School of Drug Discovery and Medical Information Science, Gifu University, Gifu, Japan

**Keywords:** Microrna-143, microRNA-145, LV dysfunction, LV dilation, Heart diseases

## Abstract

MicroRNA(miR)-143 and miR-145 are mainly expressed in vascular smooth muscle cells. However, the relationship between plasma miR-143 or miR-145 levels and the left ventricular (LV) function in patients with heart diseases remains unclear. Blood samples were taken from the antecubital vein in patients with heart diseases (*n* = 52), such as coronary artery disease, old myocardial infarction, cardiomyopathy, and valvular heart disease, and controls without heart diseases (*n* = 22). We measured plasma miR-143 and -145 levels by quantitative RT–PCR using TaqMan MicroRNA Assays and THUNDERBIRD Probe qPCR Mix. Plasma BNP levels were also measured. Echocardiography was performed to measure the LV ejection fraction (LVEF) and LV dilation. Plasma miR-143 and miR-145 levels were significantly higher in patients with heart diseases than in controls, respectively. Plasma miR-143 and miR-145 levels were significantly higher in patients with LVEF < 50% than in those with LVEF ≧ 50%, respectively. Plasma miR-143 and miR-145 levels were inversely correlated with LVEF, respectively. Plasma miR-143 and miR-145 levels were positively correlated with LV end-systolic dimension, respectively. Plasma miR-143 and -145 levels were positively correlated with plasma BNP levels, respectively. Plasma BNP levels were inversely correlated with LVEF. Plasma miR-143 and miR-145 levels are elevated in patients with LV dysfunction and may counteract LV dysfunction.

## Introduction

In accordance with the increased number of elderly people, the number of patients with heart failure is markedly increasing in Japan [1]. Heart failure is the end-stage phenotype of several heart diseases, such as coronary artery disease, old myocardial infarction, valvular heart disease, congenital heart disease, cardiomyopathy, and hypertension. The plasma brain natriuretic peptide (BNP) level is increased in patients with heart failure and is a powerful predictor of the prognosis associated with symptomatic and asymptomatic heart failure [2, 3].

The microRNAs (miRs) are 21–25 base non-coding RNAs, and approximately 2700 miRs have been identified in humans (miRBase, http://www.mirbase.org/). The miRs negatively regulate messenger RNA (mRNA) expression by inhibiting translation or degrading mRNAs [4]. It has been reported that miRs play an important role in a variety of biological processes, such as cell death, cell proliferation, and cell differentiation [5]. Recently, some of miRs have been reported to serve as a potential biomarker for heart failure [6, 7]. Among many miRs, miR-143 and miR-145 are associated with the proliferation of vascular smooth muscle cells [8] and progression of atherosclerosis [9]. It was reported that miR-143 and miR-145 are expressed in the heart [8, 10, 11], miR-143 regulates morphogenesis of the heart [11], and miR-145 regulates hypertrophy of the heart [11]. Therefore, miR-143 and miR-145 may regulate the pathophysiology of heart diseases [12]. We previously reported that the intravenous administration of miR-145 after acute myocardial infarction (AMI) reduces the myocardial infarct size and improves the left ventricular (LV) function as compared with controls in rabbits [13], and we recently reported that plasma miR-143 and miR-145 levels increase in the acute phase of AMI and that the increase in plasma miR-143 levels positively correlated with recovery of LV function and the increase in plasma miR-145 tended to positively correlate with recovery of LV function in the chronic phase at 6 months in patients with AMI [14].

Since miR-143 and miR-145 have been reported to be located approximately 1.3 kb from each other on chromosome 5q33 and have similar characteristics [15], both miR-143 and miR-145 may contribute to repair damaged cardiac tissue and improve the deteriorated LV function in patients with heart diseases. However, the behavior of plasma miR-143 and miR-145 levels and association between plasma miR-143 or miR-145 levels and LV function in the chronic state have not yet to be clarified in patients with heart diseases. Thus, in the present study, we aimed to investigate the relationship between plasma miR-143 or miR-145 levels and the LV function, between plasma miR-143 or miR-145 levels and LV dilation, and between plasma miR-143 or miR-145 levels and the plasma BNP level, a well-known marker of heart failure, in patients with heart diseases.

### Subjects and methods

The protocol of the present study was approved by the ethics committee of Gifu University Graduate School of Medicine (Approval number: 30-011) and Gifu Municipal Hospital (Approval number: 455). The investigation conformed with the principles outlined in the Declaration of Helsinki (Br Med J 1964; ii:177). The public and trial registry number is UMIN000040165.

### Study patients

This study included a total of 74 patients who underwent cardiac catheterization and echocardiography at Gifu City Hospital and Gifu University Hospital to investigate cardiac disease in patients with precordial complaints and breathing difficulties. Control group (*n* = 22) consists of patients without significant coronary artery stenosis or left ventricular damage, and some of them were previously performed percutaneous coronary artery intervention and receiving some drugs. In contrast, heart disease group (*n* = 52) consists of patients with significant coronary stenosis, old myocardial infarction, cardiomyopathy and valvular heart disease. The subjects consisted of 30 males and 44 females, with a mean age of 71.4 ± 10.7 years. The study period was from July 2018 to June 2021.

### Echocardiography

The LV ejection fraction (LVEF), LV end-diastolic dimension (LVDd), and LV end-systolic dimension (LVSd) were obtained by echocardiography (iE33, PHILIPS, Tokyo, Japan). We used the modified Simpson’s method, which is regarded as a reliable method to estimate LVEF. An echocardiologist performed the echocardiography, who was blinded to the protocol of this study. Echocardiography was performed before the day of cardiac catheterization.

### Measurements of plasma miR-143 and miR-145 levels, and plasma BNP levels

The timing of blood sampling for miR assay was on the day of cardiac catheterization. Blood samples were taken from the antecubital vein and collected into sterile tubes containing EDTA, immediately placed on ice, and then centrifuged at 1500 g for 15 min. Plasma was then collected and frozen at  – 83 °C until further analysis. To minimize RNA degradation, we only used samples that were freeze‐thawed once. MiRNAs from frozen plasma were extracted using the NucleoSpin miRNA Plasma kit (MACHEREY‐NAGEL GmbH, Düren, Germany) according to the standard protocol. Proteins in the supernatant were precipitated using a reagent in the kit and removed by centrifugation. After adjustment of the binding conditions with isopropanol, miRNAs were bound to a miRNA collection column. The miRNA quality was assessed by measuring circulating miR16‐5p levels. The purified miRNA was immediately used for reverse transcription to prevent degradation of RNA before the PCR step. To determine plasma miR-143 and miR-145 levels, we conducted quantitative RT–PCR (qRT–PCR) using TaqMan microRNA assays and THUNDERBIRD Probe qPCR Mix.

Since miR16‐5p is abundantly and constantly found in plasma of the control and heart failure patients, similar to ribosomal RNA in cellular RNAs, we used miR16-5p as an internal control. Therefore, its levels reflect miRNA degradation and the quality of plasma samples. As to this, we cited 5 papers from our laboratory and other laboratories [14, 16–19]. Plasma miR-143 and miR-145 levels are expressed relative to miR-16 as an internal control and expressed as ∆Ct. The repeatability for measurement of miRNA levels was confirmed by assessing the same samples multiple times (average coefficient of variation less than 0.25, *n* = 4) [14]. Plasma BNP levels were measured by the Shionoria BNP RIA kit (Shionogi, Osaka, Japan).

### Blood biochemical analysis

Blood samples were taken from the antecubital veins. Creatinine, total-cholesterol (TC), low-density lipoprotein cholesterol (LDL-C), high-density lipoprotein cholesterol (HDL-C), triglycerides (TG), and hemoglobin A1c (HbA1C) were measured.

### Complications and drugs used

Complications such as hypertension, diabetes mellitus, hyperlipidemia, and drugs used were examined.

### Statistical analysis

Data are shown as the mean ± standard deviation (SD). Categorical data are summarized as percentages and compared with a Chi-square test or Fisher’s exact test, as appropriate. The normality of data distributions was tested using the Kolmogorov–Smirnov test. The significance of differences between groups for variables that were normally distributed was determined by the unpaired Student’s *t* test. Correlation coefficients between two variables were obtained by linear regression analysis using Pearson’s correlation analysis. These statistical analyses were performed using GraphPad Prism 7 (GraphPad Software Inc.). A *p* value < 0.05 was considered significant, and *p* < 0.01 and *p* < 0.001 were considered highly significant. Factors for LVEF and LVSd were analyzed by using logistic regression models. Multivariate logistic regression analysis (forward selection, based on the likelihood ratio, or forced entry method) was performed. A two-sided *p* value of less than 0.05 was considered statistically significant. Statistical analysis was made with SPSS for Windows version 22.0 (IBM Japan, Tokyo, Japan).

## Results

### Patients’ characteristics and drugs used

Patients’ characteristics are shown in Table 1. In the control group (*n* = 22, male: *n* = 4, female: *n* = 18), the mean age was 73.9 ± 7.5 years. In the heart disease group (*n* = 52, male: *n* = 26 female: *n* = 26), the mean age was 70.6 ± 11.5 years. Heart diseases consisted of coronary artery disease (*n* = 19), old myocardial infarction (*n* = 23), valvular heart disease (*n* = 5), cardiomyopathy (*n* = 2), and arrhythmias (*n* = 3), and controls were subjects without heart diseases (*n* = 22). Biochemical data are shown in Table 1. Drugs used were ACE I/ARBs (*n* = 39), CCBs (*n* = 33), beta-blockers (*n* = 30), statins (*n* = 41), insulin (*n* = 4), DPP4 inhibitors (*n* = 15), SGLT2 inhibitors (*n* = 5), Metoformin (*n* = 11), antiplatelets (*n* = 42), and DOAC (*n* = 9). Complications were hypertension (*n* = 49), dyslipidemia (*n* = 35), and diabetes mellitus (*n* = 32).

**Table 1:** Patient’s characteristics and drugs used

	Control group (*n* = 22)	Cardiac disease group (*n* = 52)	*P* value
Age, years	73.9 ± 7.5	70.6 ± 11.5	0.1552
Sex, (*n*)	M/F, 4/18	M/F, 26/26	0.0185
*Subjects*, *n*			
HTN, *n*(%)	17 (77.3)	32 (61.5)	0.2827
HL, *n*(%)	11 (50.0)	24 (46.2)	0.8032
DM, *n*(%)	8 (36.4)	24 (46.2)	0.6083
*Biochemical data*			
Creatinine (mg/dL)	1.03 ± 0.96	0.92 ± 0.36	0.6133
TC (mg/dL)	181 ± 26.4	181 ± 36.6	0.9898
LDL-C (mg/dL)	102 ± 19.1	104 ± 32.4	0.83
HDL-C (mg/dL)	48.9 ± 14.3	53.9 ± 16.5	0.221
TG (mg/dL)	138 ± 62.0	156 ± 109	0.3929
HbA1c (%)	6.2 ± 0.71	6.4 ± 0.91	0.6601
Drug used, *n*(%)			
ACEI/ARB	9 (40.9)	30 (57.7)	0.2116
CCB	15 (68.2)	18 (34.6)	0.0107
Beta blocker	7 (31.8)	23 (44.2)	0.4383
Statin	12 (54.5)	26 (50.0)	0.8019
Insulin	1 (4.5)	3 (5.8)	0.831
DPP4-Inhibitor	3 (13.6)	12 (23.1)	0.5293
SGLT2-Inhibitor	0 (0)	5 (9.6)	0.3133
Metformin	4 (18.2)	7 (13.5)	0.7227
Antiplatelet	12 (54.5)	30 (57.7)	0.8038
DOAC	2 (9.1)	7 (13.5)	0.599

*HTN*  hypertension, *HL*  hyperlipidemia, *DM*  diabetes mellitus, *TC*  total cholesterol, *LDL-C*  low density lipoprotein cholesterol, *HDL-C*  high density lipoprotein cholesterol, *TG*  triglyceride, *CCB*  calcium channel blocker, *DOAC*  direct oral anticoagulant

### Plasma miR-143 and miR-145 levels in controls and heart disease patients

**Fig. 1:**
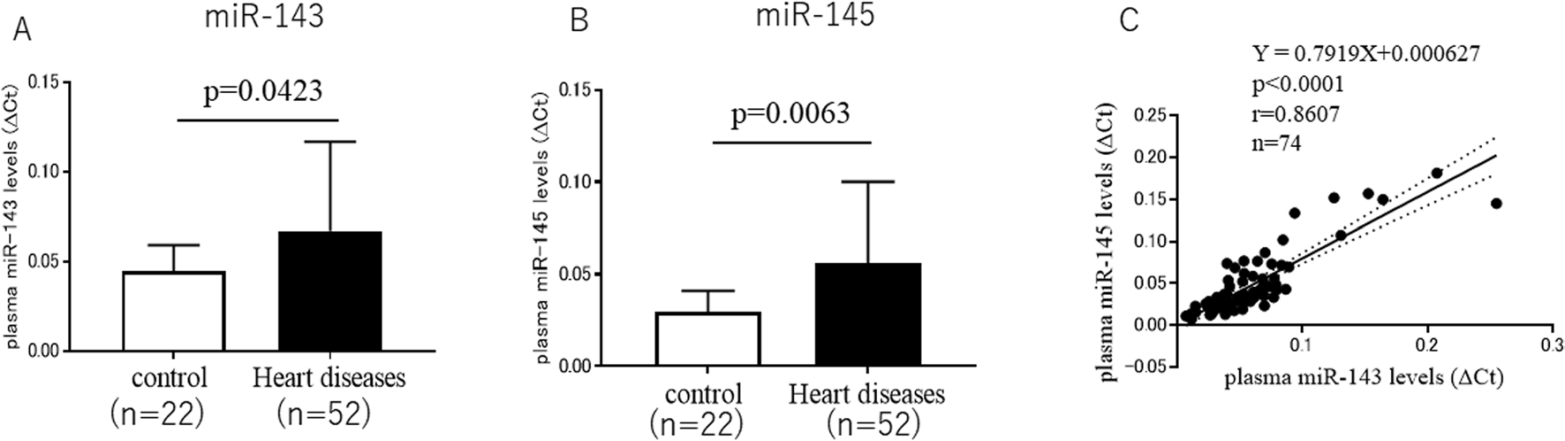
**A** Plasma miR-143 levels in control and heart disease groups. **B** Plasma miR-145 levels in control and heart disease groups. **C** Relationship between plasma miR-143 levels and plasma miR-145 levels

Plasma miR-143 levels were significantly higher in the heart disease group than in the control group (*p* = 0.0423) (Fig. 1A). Plasma miR-145 levels were also significantly higher in the heart disease group than in the control group (*p* = 0.0063) (Fig. 1B).

### Relationship between plasma miR-143 levels and plasma miR-145 levels

Plasma miR-143 levels were closely and positively correlated with plasma miR-145 levels (Fig. 1C).

### Plasma miR-143 and miR-145 levels between LVEF≧50% and LVEF < 50%

**Fig. 2:**
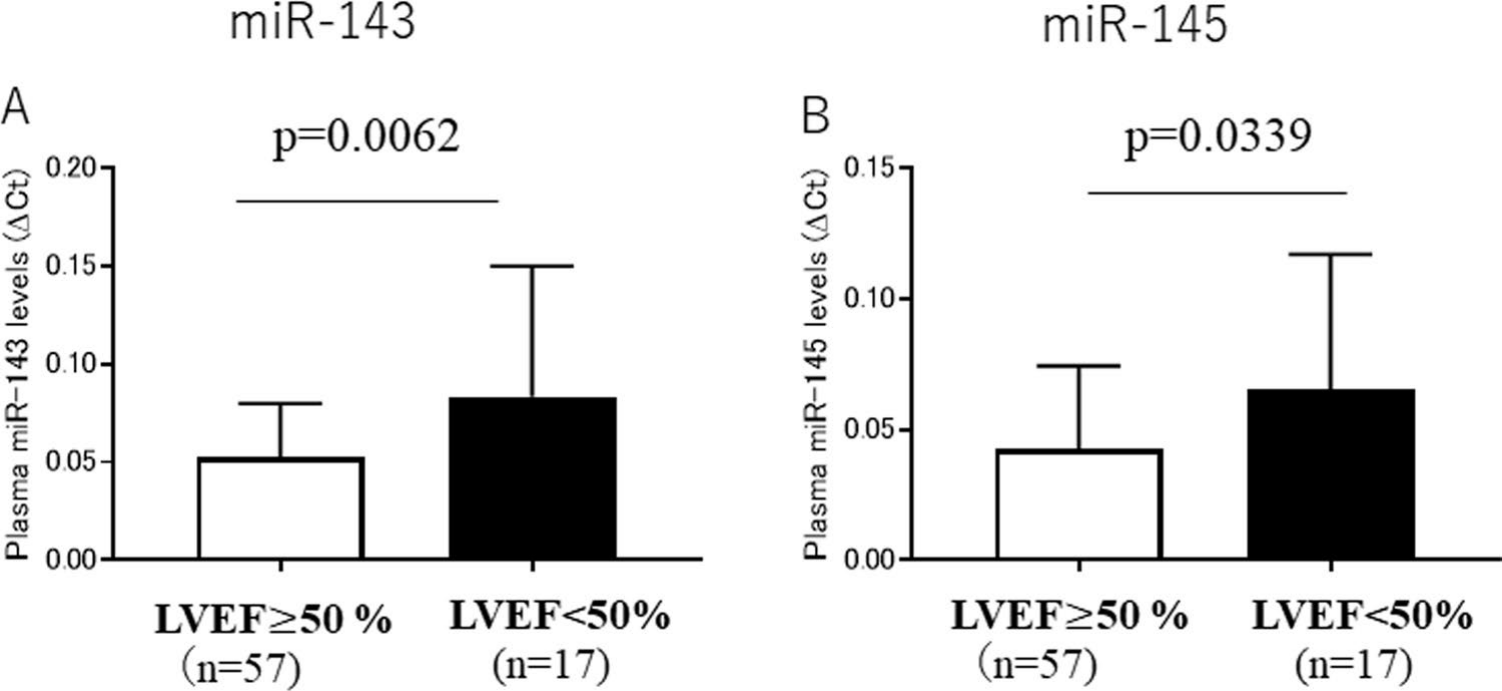
**A** Plasma miR-143 levels in LVEF≧50% and < 50% groups. **B** Plasma miR-145 levels in LVEF≧50% and < 50% groups

Plasma miR-143 levels were significantly higher in the LVEF < 50% group than in the LVEF**≧**50% group (*p* = 0.0062) (Fig. 2A). Plasma miR-145 levels were also significantly higher in the LVEF < 50% group than in the LVEF**≧**50% group (*p* = 0.0339) (Fig. 2B).

### Relationship between plasma miR-143 or miR-145 levels and LVEF

**Fig. 3:**
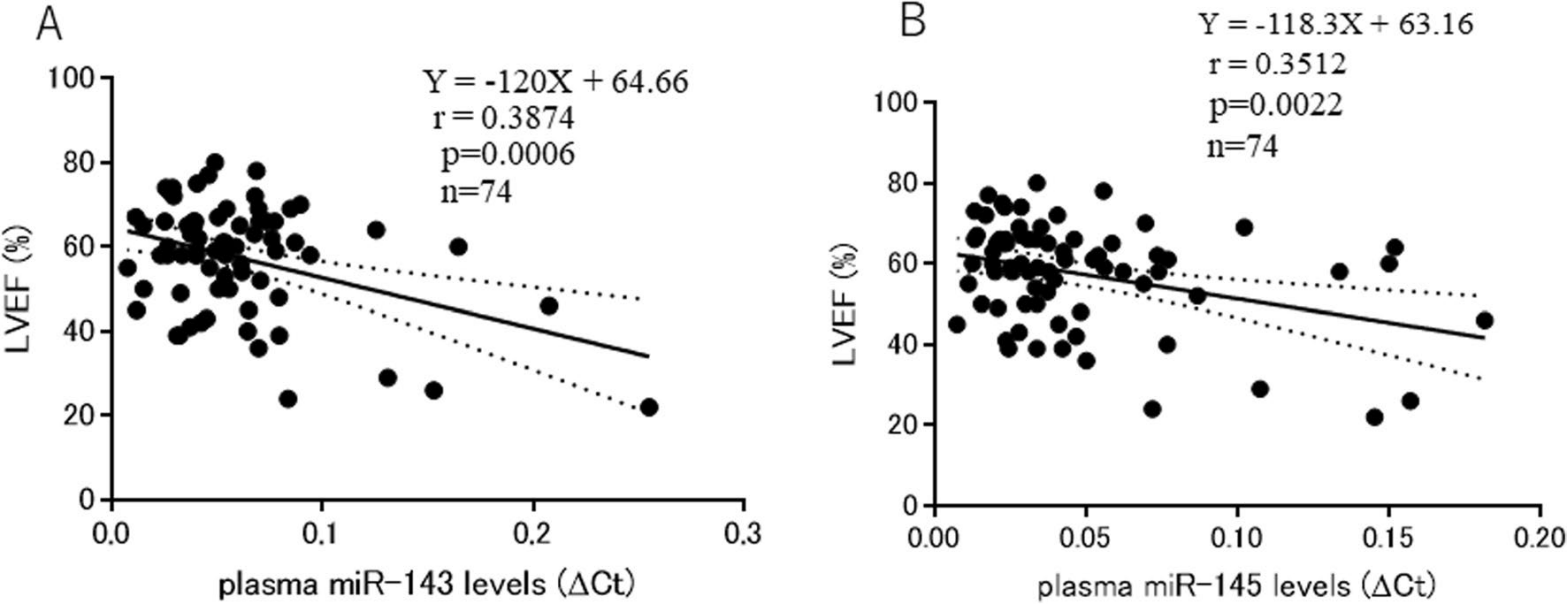
**A** Relationship between plasma miR-143 levels and LVEF. **B** Relationship between plasma miR-145 levels and LVEF

Plasma miR-143 levels were inversely correlated with LVEF (*n* = 74, *r* = 0.3874, *p* = 0.0006) (Fig. 3A). Plasma miR-145 levels were also inversely correlated with LVEF (*n* = 74, *r* = 0.3512, *p* = 0.0022) (Fig. 3B).

### Relationship between plasma miR-143 or miR-145 levels and LV dilation

**Fig. 4:**
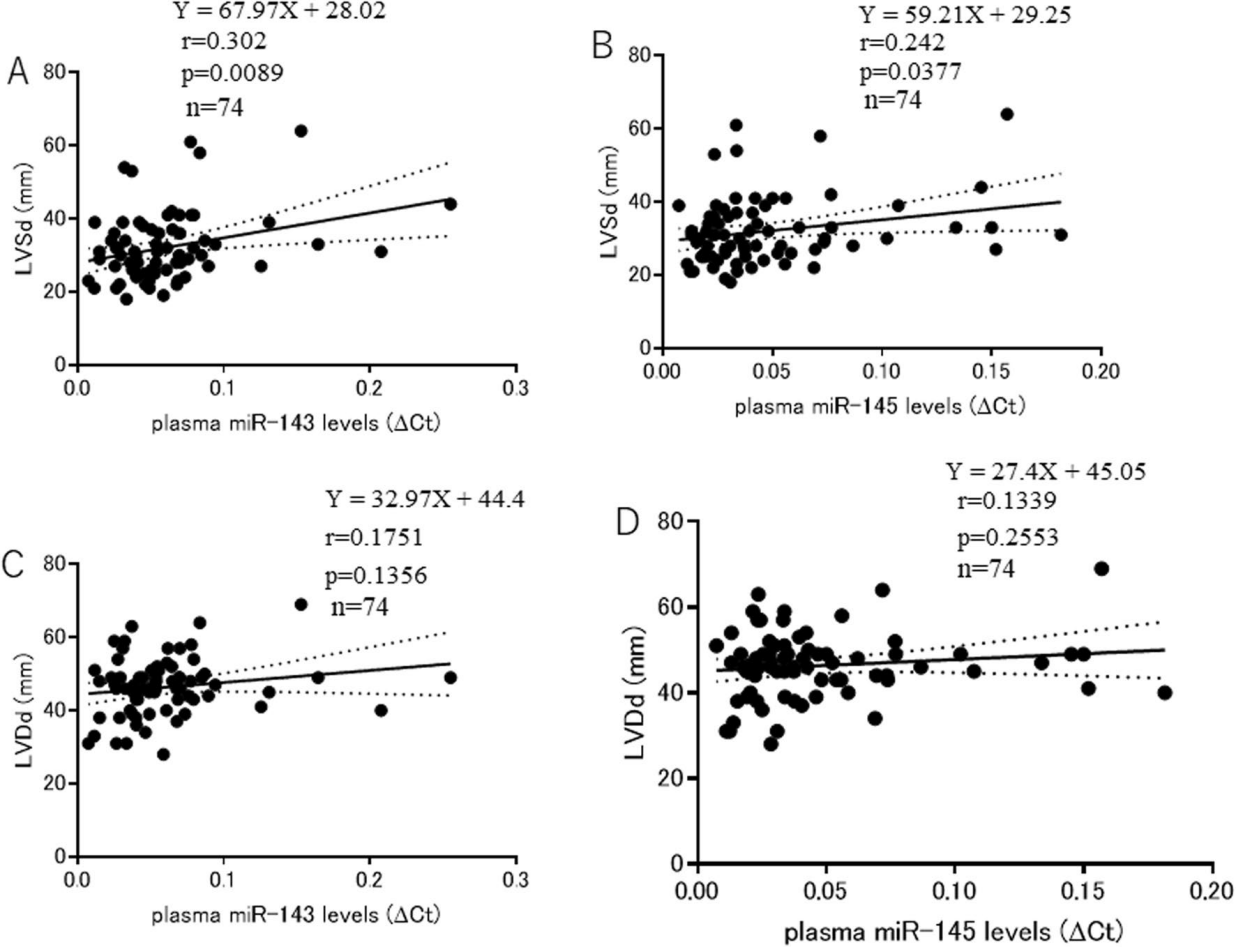
The **A** Relationship between plasma miR-143 levels and LVSd. **B** Relationship between plasma miR-145 levels and LVSd. **C** Relationship between plasma miR-143 levels and LVDd. **D** Relationship between plasma miR-145 levels and LVDd

Plasma miR-143 levels were positively correlated with the LV end-systolic dimension (LVSd), an indicator of LV remodeling (*n* = 74, *r* = 0.302, *p* = 0.0089) (Fig. 4A). The plasma miR-145 levels were also positively correlated with LVSd (*n* = 74, *r* = 0.242, *p* = 0.0377) (Fig. 4B). Plasma miR-143 levels were not correlated with the LV end-diastolic dimension (LVDd) (*n* = 74, *r* = 0.1751, *p* = 0.1356) (Fig. 4C). Plasma miR-145 levels were not correlated with LVDd (*n* = 74, *r* = 0.1339, *p* = 0.2553) (Fig. 4D).

### Relationship between plasma BNP levels and LVEF, plasma miR-143 levels, or plasma miR-145 levels

**Fig. 5:**
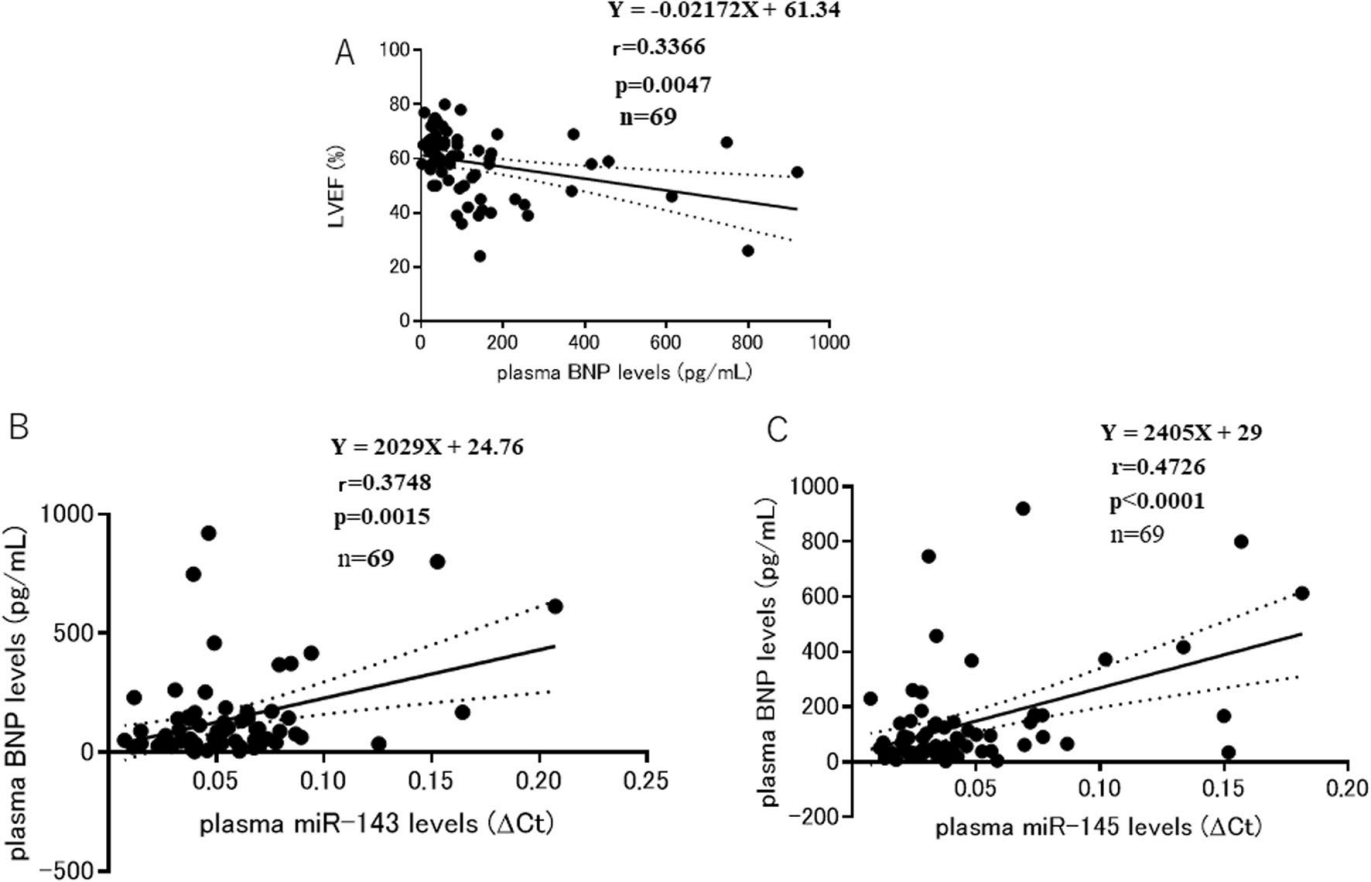
The **A** Relationship between plasma BNP levels and LVEF. **B:** Relationship between plasma miR-143 and plasma BNP levels. **C:** Relationship between plasma miR-145 and plasma BNP levels

Plasma BNP levels were inversely correlated with LVEF (*n* = 69, *r* = 0.3366, *p* = 0.0047) (Fig. 5A). Plasma miR-143 levels were positively correlated with plasma BNP levels (*n* = 69, *r* = 0.3748, *p* = 0.0015) (Fig. 5B). Plasma miR-145 levels were also positively correlated with plasma BNP levels (*n* = 69, *r* = 0.4726, *p* < 0.0001) (Fig. 5C).

### Factors that may affect LVEF and LVSd

Many of the factors that may affect LVEF and LVSd were compared between the LVEF ≧50% and LVEF < 50% groups and between LVSd > 42 mm and LVSd≦42 mm groups (Tables 2, 3). Among many factors, univariate analysis showed a significant difference in miR-143 (*p* = 0.0062) and miR-145 (*p* = 0.0339) for LVEF (Table 2) and showed a significant difference in miR-143 (*p* = 0.0034) and miR-145 (*p* = 0.0469) for LVSd (Table 3). Multivariate logistic regression analysis showed a significant difference (OR = 1.178, 95% CI 1.025–1.355, *p* = 0.021) in miR143 for LVEF (Table 2), and showed a significant difference (OR = 1.196, 95% CI 1.030–1.389, *p* = 0.019) in miR-143 for LVSd (Table 3). Therefore, among many factors, only miR-143 was more strongly correlated with LVEF and LVSd than miR-145.

**Table 2:** Comparison of factors that affect LVEF

	LVEF≧50% (*n* = 57)	LVEF < 50% (*n* = 17)	*P* value
miR-143 (pg/ml)	0.0526 ± 0.0273	0.0835 ± 0.0667	0.0062
miR-145 (pg/ml)	0.0428 ± 0.0317	0.0650 ± 0.0521	0.0339
*Characteristics*			
Age (years)	73 ± 10.6	74 ± 11.2	0.7152
Sex, (*n*)	M/F, 23/34	M/F, 7/10	1
HTN, *n*(%)	41 (71.9)	8 (47.1)	0.0801
HL, *n*(%)	28 (49.1)	7 (47.1)	0.593
DM, *n*(%)	22 (38.6)	10 (58.8)	0.1695
*Biochemical data*			
Creatinine (mg/dL)	0.95 ± 0.67	0.97 ± 0.32	0.893
TC (mg/dL)	181 ± 33.7	182 ± 33.5	0.9689
LDL-C (mg/dL)	105 ± 29.2	100 ± 32.3	0.6732
HDL-C (mg/dL)	53.9 ± 17.2	46.2 ± 9.4	0.1145
TG (mg/dL)	144 ± 70.0	170 ± 159	0.3567
HbA1c (%)	6.25 ± 0.84	6.57 ± 0.92	0.2153
*Medications used, n*(%)			
ACEI/ARB	30 (52.6)	9 (52.9)	1
CCB	31 (54.4)	2 (11.8)	0.002
Beta blocker	21 (36.8)	9 (52.9)	0.2696
Statin	30 (52.6)	8 (47.1)	0.785
Insline	3 (5.3)	1 (5.9)	1
DPP4-Inhibitor	10 (17.5)	5 (29.4)	0.313
SGLT2-Inhibitor	2 (3.5)	3 (17.6)	0.0762
Metphrmine	10 (17.5)	1 (5.9)	0.4385
Antiplatelet	33 (57.9)	9 (52.9)	0.7842
DOAC	5 (8.8)	4 (23.5)	0.1972

*HTN*  hypertension, *HL*  hyperlipidemia, *DM*  diabetes mellitus, *TC*  total cholesterol, *LDL-C*  low density lipoprotein cholesterol, *HDL-C*  high density lipoprotein cholesterol, *TG*  triglyceride, *CCB* calcium channel blocker, *DOAC*  direct oral anticoagulant

**Table 3:** Comparison of factors that affect LVSD

	LVSd≦42 mm (*n* = 68)	LVSd > 42 mm (*n* = 6)	*P* value
miR-143 (pg/ml)	0.0556 ± 0.0453	0.1063 ± 0.0847	0.0034
miR-145 (pg/ml)	0.0453 ± 0.0351	0.0774 ± 0.0595	0.0469
*Characteristics*			
Age (years)	73 ± 10.4	71 ± 13.7	0.422
Sex, (*n*)	M/F, 26/42	M/F, 4/2	0.2151
HTN, *n*(%)	47 (69.1)	2 (33.3)	0.1708
HL, *n*(%)	32 (47.1)	3 (50.0)	1
DM, *n*(%)	29 (42.6)	3 (50.0)	1
*Biochemical data*			
Creatinine (mg/dL)	0.94 ± 0.62	1.13 ± 0.38	0.469
TC (mg/dL)	180 ± 33.2	194 ± 36.4	0.379
LDL-C (mg/dL)	103 ± 29.7	110 ± 32.9	0.692
HDL-C (mg/dL)	52.2 ± 16.6	53.0 ± 7.97	0.917
TG (mg/dL)	148 ± 99.3	169 ± 81.6	0.633
HbA1c (%)	6.31 ± 0.85	6.40 ± 1.11	0.817
*Medications used*, *n*(%)			
ACEI/ARB	35 (51.5)	4 (66.7)	0.677
CCB	33 (48.5)	0 (0)	0.0303
Beta blocker	27 (39.7)	3 (50.0)	0.6812
Statin	35 (51.5)	3 (50.0)	1
Insline	4 (5.9)	0 (0)	1
DPP4-Inhibitor	13 (19.1)	2 (33.3)	0.5946
SGLT2-Inhibitor	4 (5.9)	1 (16.7)	0.3529
Metphrmine	11 (16.2)	0 (0)	0.5826
Antiplatelet	40 (58.8)	2 (33.3)	0.3929
DOAC	8 (11.8)	1 (16.7)	0.5541

*HTN*  hypertension, *HL*  hyperlipidemia, *DM*  diabetes mellitus, *TC*  total cholesterol, *LDL-C*  low density lipoprotein cholesterol, *HDL-C*  high density lipoprotein cholesterol, *TG*  triglyceride, *CCB*  calcium channel blocker, *DOAC*  direct oral anticoagulant

## Discussion

The findings of the present study were that: (1) plasma miR-143 and miR-145 levels were significantly higher in patients with heart diseases than controls, respectively, (2) plasma miR-143 and miR-145 levels were significantly higher in patients with LVEF < 50% than in those with LVEF ≧ 50%, respectively, (3) plasma miR-143 and miR-145 levels were inversely correlated with LVEF, respectively, (4) plasma miR-143 and miR-145 levels were positively correlated with the LV end-systolic dimension (LVSd), respectively, (5) plasma BNP levels were positively correlated with LVEF, and (6) plasma miR-143 and miR-145 levels were positively correlated with plasma BNP levels, respectively.

It has been reported that miR-143 and miR-145 are involved in the pathogenesis of many cardiovascular diseases, such as essential hypertension [20, 21], atherosclerosis [9, 22, 23], coronary artery disease [24, 25], and myocardial infarction [13]. MiR-143 and miR-145 are highly expressed in vascular smooth muscle cells [8, 23, 26] and the heart [8, 10]. MiR-143 and miR-145 are present in the peripheral blood in the form of a nuclease-resistant complex with argonaute (AGO) protein [27] or in exosomes [28] released from the cells containing miR-143 and miR-145, such as vascular smooth muscle cells and cardiomyocytes. However, the behavior of plasma miR-143 and -145 levels in cardiac patients with LV dysfunction has not still been fully clarified. Therefore, the measurement of plasma levels of miR-143 and miR-145 may help to understand the roles of miR-143 and miR-145 in the pathophysiology of LV dysfunction in patients with heart diseases. As a matter of fact, other studies reported that miR-143 and miR-145 were associated with cardiovascular diseases [29] and that plasma miR-143 and miR-145 levels increased in patients with advanced heart failure [30].

In the present study, plasma miR-143 and miR-145 levels were significantly higher in the heart disease group than in the control group, respectively (Fig. 1A, B). Plasma miR-143 and miR-145 levels were significantly higher in patients with LVEF < 50% than in those with LVEF**≧**50%, respectively (Fig. 2A, B), suggesting that the LV function is a determinant factor of plasma miR-143 and miR-145 levels; patients with LV dysfunction show higher plasma miR-143 and miR-145 levels, and those with a normal LV function show lower plasma miR-143 and miR-145 levels. Furthermore, we investigated the relationship between plasma miR-143 levels or plasma miR-145 levels and LVEF. As a result, plasma miR-143 and miR-145 levels were inversely correlated with LVEF, respectively (Fig. 3A, B). These results suggest that plasma miR-143 and miR-145 levels increase depending on the severity of deterioration of LV function; lower plasma miR-143 and miR-145 levels were associated with a normal LV function, and higher plasma miR-143 and miR-145 levels were associated with a deteriorated LV function.

Regarding LV chamber dilation, plasma miR-143 and miR-145 levels were positively correlated with the LV end-systolic dimension (LVSd) (*p* = 0.0089 and *p* = 0.0037, respectively) (Fig. 4A, B). However, there was no correlation between plasma 143 levels or plasma miR-145 levels and the LV end-diastolic dimension (LVDd) (Fig. 4C, D). These results suggest that plasma miR-143 and miR-145 levels increase depending on the dilation of LVSd: lower plasma miR-143 and miR-145 levels were associated with smaller LVSd, and higher plasma miR-143 and miR-145 levels were associated with greater LVSd. Since LVSd has been reported to be a good indicator of LV remodeling [31], plasma miR-143 and miR-145 levels may be associated with LV remodeling. We previously reported that the intravenous administration of miR-145 reduced the infarct size, improved the cardiac function, and attenuated LV remodeling in a rabbit model of acute myocardial infarction [13]. Since it has been reported that miR-143 and miR-145 are located approximately 1.3 kb from each other on chromosome 5q33 and have similar characteristics [15], both miR-143 and miR-145 might have similar abilities to repair damaged cardiac tissue, improve the deteriorated LV function, and attenuate LV remodeling in patients with LV dysfunction. According to the results of the animal experiment and assumption that miR-143 and miR-145 have similar effects on the heart, LV dysfunction and LV remodeling by themselves might have facilitated the release of endogenous miR-143 and miR-145 into the peripheral blood from cells in cardiac and vascular tissues, leading to the increase in plasma miR-143 and miR-145 levels.

The plasma BNP level is regarded as a diagnostic and prognostic marker of symptomatic and asymptomatic heart failure [2, 3]. In the present study, plasma BNP levels were inversely correlated with LVEF, an indicator of the LV function (Fig. 5A), suggesting that higher plasma BNP levels are associated with lower LVEF, and lower plasma BNP levels are associated with higher LVEF.

In the present study, the behaviors of plasma BNP, miR-143, and miR-145 levels were similar in terms of the relationship with LVEF. Plasma BNP, miR-143, and miR-145 levels were inversely correlated with LVEF (Figs 3A, B, 5A). In addition, there was a positive correlation between plasma BNP and miR-143 levels (*p* = 0.0015) (Fig. 5B), and between plasma BNP and miR-145 levels (*p* < 0.0001) (Fig. 5C). These results suggest that plasma miR-143 and miR-145 levels may be also diagnostic and prognostic markers of heart failure. BNP by itself has been reported to improve the cardiac function in a rat model of heart failure [32]. Angiotensin receptor–neprilysin inhibitor (ARNI), a combination of sacubitril and valsartan, is a new drug for heart failure. The PARADIGM–HF trial, in which 8,442 heart failure patients were enrolled in a double-blind and randomized trial, demonstrated that ARNI was superior to enalapril in reducing the risks of death and hospitalization for heart failure [33]. In the PARADIGM–HF trial, the blockade of neprilysin by sacubitril reduced the degradation of natriuretic peptides, resulting in an increase of plasma BNP levels [34]. Based on these studies, elevated plasma BNP levels in heart failure patients are considered to counteract LV dysfunction.

Because the present study is a clinical study demonstrating the relationship between plasma miR-143 or miR-145 levels and LV dysfunction or LV dilation, it is difficult to show the precise mechanistic insight how miR-143 and miR-145 are upregulated in heart diseases in the clinical setting. However, it has been reported that miR-143 and miR-145 can be upregulated in endothelial cells in response to shear stress and subsequently exported in exosome-like vesicles that regulate vascular smooth muscle phenotype [35]. Furthermore, in the present study, plasma miR-143 or miR-145 levels positively correlated with plasma BNP levels (Fig. 5B, C). Since BNP is regarded as a marker of intravascular volume, and BNP secretion has a direct linear correlation with intravascular volume status [36], higher plasma BNP levels mean higher intravascular volume status, leading to a greater shear stress to the vascular endothelial cells, and then upregulation of miR-143 and miR-145. Furthermore, since plasma miR-143 levels were closely and positively correlated with plasma miR-145 levels in the present study (Fig. 1C), plasma miR-143 and miR-145 levels might have behaved similarly in heart diseases due to the shear stress to the vascular endothelial cells. Therefore, one of the mechanisms by which both plasma miR-143 and miR-145 levels are elevated in patients with LV dysfunction may be related to the higher plasma BNP levels in patients with LV dysfunction.

It was previously reported that plasma miR-143 levels were increased in patients with heart failure via induction by HIF-1 [37], a direct stimulator for BNP induction [38]. This report may explain the result in the present study that higher plasma BNP levels were positively correlated with higher plasma miR-143 levels in patients with heart diseases. In the present study, however, both plasma miR-143 and miR-145 levels were significantly higher in patients with heart diseases than those in the controls. Since plasma miR-143 levels positively and closely correlated with plasma niR-145 levels (Fig. 1C), it is reasonable to consider that plasma miR-143 and miR-145 levels behave similarly in patients with heart diseases as shown in the present study.

Furthermore, we recently reported in a clinical study that plasma miR-143 and miR-145 levels increased in the acute phase (within 1 week) of acute myocardial infarction and the increase in plasma miR-143 levels in the acute phase were positively correlated with the increase in LVEF in the chronic phase of 6 months, and the increase in plasma miR-145 levels in the acute phase tended to be positively correlated with the increase in LVEF in the chronic phase of 6 months in patients with acute myocardial infarction [14]. These results mentioned above suggest that higher plasma miR-143 and miR-145 levels may improve LV function and may counteract LV dysfunction in patients with heart diseases. Furthermore, among many factors, univariate analysis demonstrated that miR-143 and miR-145 were correlated with LVEF and LVSd (Tables 2, 3).

However, multivariate logistic regression analysis demonstrated that only miR-143 was correlated with LVEF and LVSd, suggesting that miR-143 was more strongly correlated with LVEF and LVSd than miR-145.

In the present study, the target molecules for miR-143 and miR-145 in patients with heart diseases have not been clarified. However, in an acute myocardial infarction model, it has been reported that the mechanisms by which miR-145 improves the cardiac function involving acceleration of autophagy of cardiomyocytes through targeting fibroblast growth factor receptor substrate 2 (FRS2) [13], and that the mechanism by which miR-143 improves cardiac function is through decreasing oxidative stress that causes autophagy cell death by silencing COX-1, COX-2 and ATG7 [14]. The other study demonstrated the CHK2/Beclin2 pathway as a target molecule to avoid autophagic cell death induced by ischemic heart disease [39].

In conclusion, plasma miR-143 and miR-145 levels increase in heart diseases patients with LV dysfunction. Plasma miR-145 and -143 may counteract LV dysfunction.

## Data Availability

The deidentified participant data will not be shared.
